# Escaping the metric-driven governance trap in global health: how DALYs and coverage indicators mislead policymakers in LMICs

**DOI:** 10.1186/s12939-026-02909-9

**Published:** 2026-06-04

**Authors:** Yusuf Hared Abdi, Sharmake Gaiye Bashir, Yakub Burhan Abdullahi, Yusuff Adebayo Adebisi, Don Eliseo III Lucero-Prisno

**Affiliations:** 1 Faculty of Medicine and Health Science, Hormuud University, Mogadishu, Somalia; 2 College of Social Sciences, University of Glasgow, 40 Bute Gardens, Glasgow, G12 8RT UK; 3 Department of Global Health and Development, Faculty of Public Health and Policy, London School of Hygiene and Tropical Medicine, London, UK; 4 Office for Research, Extension and Innovations, Bukidnon State University, Malaybalay City, Bukidnon, Philippines; 5 Research Office, Palompon Institute of Technology, Palompon, Leyte, Philippines

**Keywords:** Global health metrics, Disability-adjusted life years (DALYs), Health governance, Epistemic justice, Low- and middle-income countries (LMICs)

## Abstract

Every year, billions of dollars in global health financing are allocated on the basis of a single number a disability-adjusted life year, a coverage rate, or a composite index generated thousands of miles from the communities that will bear the consequences of those decisions. The structural disconnect between the production of global health metrics and their application in low- and middle-income countries (LMICs) is not merely a technical inconvenience; it is a defining feature of an unequal international order. As LMIC governments face mounting pressure to demonstrate accountability to donors and multilateral institutions, the metrics that ostensibly measure their progress have come to shape not only what is measured but what is funded, prioritized, and ultimately valued. We argue that the dominant tools of global health measurement disability-adjusted life years (DALYs), quality-adjusted life years (QALYs), coverage indicators, and composite indices systematically misrepresent health realities in LMICs, distort policy priorities, and actively undermine the health system strengthening. Our argument is not that coverage indicators are without value measuring whether people access life-saving interventions remains vital but that their deployment as the primary grammar of global health governance, uncoupled from system-level and equity dimensions, actively distorts decision-making. This is not simply a technical problem of imperfect indicators; it is a metric-driven governance trap that locks LMICs into externally defined priorities and distorts the politics of health decision-making. We argue that escaping this trap requires a fundamental reorientation of the global health measurement enterprise: one that redirects investment from international modelling to national data systems, subjects the normative assumptions embedded in global metrics to genuinely participatory revision, and institutionalises locally driven measurement frameworks co-designed with LMIC governments, civil society, and communities. Only by moving methodological authority and analytical sovereignty closer to where illness is actually lived can global health metrics become tools of accountability rather than instruments of abstraction.

## The architecture of global health measurement

The contemporary architecture of global health metrics was largely established by the 1993 World Bank *World Development Report: Investing in Health*, which introduced the DALY as a novel composite indicator designed to quantify population health loss by combining years of life lost to premature mortality with years lived with disability [1]. The appeal was conceptually powerful: a single, universally applicable unit that would permit comparisons across diseases, populations, and geographies, thereby enabling rational, cost-effective priority setting in resource-limited settings [2]. The Global Burden of Disease (GBD) study, subsequently institutionalised within the Institute for Health Metrics and Evaluation (IHME), extended this framework across 281 diseases and injuries and 67 risk factors, generating estimates for virtually every country in the world [3]. QALYs, developed in parallel within health economics, provided a similar amalgamation of survival and health-related quality of life for use in cost-effectiveness analyses [4]. Coverage indicators—rates of vaccination, antenatal care uptake, skilled birth attendance, and antiretroviral treatment—followed as operationally convenient proxies for progress towards the Millennium Development Goals and, subsequently, the Sustainable Development Goals [2]. Together, these tools became the lingua franca of global health governance, structuring donor reporting requirements, funding eligibility criteria, and international rankings of health system performance [3]. Their ascendancy was accompanied by genuine achievements. The quantification of disease burden drew political attention to neglected conditions, most notably mental illness, which mortality-based statistics had consistently marginalised. The systematic cataloguing of risk-factor contributions to premature death has informed tobacco control, dietary guidelines, and air quality standards in multiple regions. Yet the consolidation of these metrics as the primary grammar of global health policy brought a set of structural limitations that became particularly consequential in LMIC contexts limitations that the global health community has been too slow to acknowledge and too reluctant to address [5].

## The fragile foundations of global estimates

The single greatest epistemic problem with global health metrics as applied to LMICs is that they rest on a foundation of data that is, in large parts of the world, absent, incomplete, or unreliable [6]. In sub-Saharan Africa, disease-specific routine health data of acceptable quality are commonly unavailable, and data collected through health facility reporting systems are rarely complete or representative of the most vulnerable segments of the population [7]. Studies assessing routine health information systems in West Africa have documented severe discrepancies between digitally reported and facility-level data, with one Nigerian analysis of maternal health records recording a Cohen’s kappa coefficient of merely 0.1036 a statistic that measures inter-rater or inter-system agreement on a scale from 0 (agreement no better than chance) to 1 (perfect agreement), indicating near-random concurrence between reporting platforms [7]. The implication is stark: the numbers that enter global estimation models are not reliable empirical measurements; they are, at best, informed approximations [6].

Institutions such as IHME have responded to this by deploying sophisticated statistical modelling using covariates (additional variables incorporated into a model to account for factors that could otherwise confound the relationship of interest), hierarchical regression (a technique that estimates values for units nested within larger groups, such as districts within countries, borrowing strength from the group when individual-level data are sparse), and data synthesis from diverse and often non-representative sources to produce national and subnational estimates, even where primary data are scarce [8]. When two independent research teams applying comparable methods to the same disease, tuberculosis, arrive at substantially different burden estimates, this signals not only methodological failure but also a fundamental constraint: models built on sparse and heterogeneous inputs produce wide uncertainty intervals that are routinely stripped away before estimates reach policymakers’ desks [8]. The WHO and IHME estimates for under-five mortality in several regions have diverged by 15–20%, with materially different epidemiological assumptions driving each set of projections [9]. Rather than expressing the fundamental limitations of surveillance infrastructure, these models communicate false precision an authoritativeness that country-level data cannot support [6].

## How metrics distort policy

The consequences of this reliance on modelled estimates are not merely academic [10]. This configuration of incentives, where what is counted becomes what is funded, constitutes a metric-driven governance trap, in which indicators rather than people quietly become the primary objects of policy. When the dominant metrics of disease burden prioritize conditions where quantification is technically tractable, they systematically disadvantage conditions where the burden is diffuse, social in origin, or resistant to discrete measurement [5]. Mental health, multimorbidity, chronic pain, palliative care needs, and the structural determinants of ill health poverty, gender-based violence, housing insecurity do not register cleanly in DALY calculations and are correspondingly under-represented in the disease control packages that DALYs justify [10].

This critique must be stated with precision. There is compelling, high-quality evidence that expanding access to antiretroviral therapy, routine childhood vaccination, and quality antenatal and postnatal care substantially reduces avertable premature mortality; in all settings, regardless of income level, these goals remain ethically and epidemiologically imperative. The concern raised here is not that such coverage should be unmeasured, but that using these indicators *alone* divorced from the strength of the health system that delivers them, the equity of access across population subgroups, and the quality of the care provided creates perverse incentives. Health systems may optimize the indicator rather than the underlying health outcome [2]. Governments competing for access to performance-based financing divert managerial attention and administrative capacity towards meeting reportable targets while neglecting the structural investments—human resources, supply chain integrity, community trust, and referral linkages that determine whether a measured coverage rate translates into genuine health improvement [11]. The proliferation of vertical disease-specific programmes, each with its own reporting requirements, indicators, and accountability mechanisms, has fragmented health system management in ways that routine coverage metrics fail to capture and actively incentivize [12]. Studies from Mozambique document how the rapid scale-up of vertical HIV/AIDS funding justified partly by DALY-based cost-effectiveness evidence diverted scarce health workers and infrastructure from primary care systems, with cascading effects on maternal and child health outcomes that fell entirely outside the metrics being monitored [13]. Across many LMICs, including fragile and conflict-affected settings in East Africa, ministerial briefings and investment cases now routinely begin with external burden estimates and coverage dashboards, even when national statisticians and programme managers privately doubt their validity.

The phenomenon is not unique to HIV [11]. India’s experience with successive disease-focused vertical programmes endorsed on the basis of cost-effectiveness analyses conducted in international centres illustrates how metric-driven priority setting can entrench a selective primary care model that leaves health systems poorly prepared for the epidemiological complexity they confront. Decades of targeted investment in discrete disease programmes, each justifiable on narrow cost-effectiveness grounds, produced measurable gains in programme-specific coverage while chronically underfunding the general primary care infrastructure, human resource systems, and surge capacity upon which broader health security depends. When COVID-19 struck, these structural deficits were immediately exposed: hospitals ran out of oxygen and beds, rural areas lacked basic diagnostic facilities, and the pre-existing shortage of a generalist health workforce crowded out by vertical programme staffing severely constrained the country’s pandemic response. The coverage metrics that had tracked India’s disease programme performance had remained silent on these systemic vulnerabilities, which were visible only once a shock arrived that could not be addressed by any single vertical programme [14]. The Ebola epidemic of 2014–2016 exposed precisely this systemic fragility in Guinea, Liberia, and Sierra Leone: health systems whose coverage statistics looked, by international standards, adequate, but whose functional capacity for surge response, laboratory diagnostics, and community engagement had been chronically starved of investment in favor of vertical programmes with demonstrable DALY returns [11]. When the metric is optimized, the system behind it can simultaneously collapse [11].

The disability weighting system embedded within the DALY calculation introduces further distortion. The original disability weights underpinning the 1990 Global Burden of Disease study were derived from a deliberative exercise conducted with a panel of health professionals predominantly from high-income countries who evaluated 22 indicator conditions using a ‘person trade-off’ technique and then classified the remaining disease sequelae by analogy. Critics noted, with considerable force, that the lived experience of disability in populations across Africa, South Asia, and Latin America was entirely absent from this process: weights for conditions borne overwhelmingly by people in LMICs were set by experts in high-income settings who had no direct experience of those conditions. In response to these criticisms, IHME undertook a major revision for the GBD 2010 study, incorporating population-based surveys in which lay respondents including some from LMICs compared hypothetical health states, producing a new set of weights intended to reflect broader societal rather than purely expert preferences. While this represented a genuine methodological advance, subsequent research has shown that the revised weights continue to diverge substantially from preferences elicited in low-income, community-based settings: a household survey in Cape Town found that locally derived disability weights differed significantly from the GBD 2010 universal set, raising questions about whether any single global set can validly represent the diverse ways that disability is experienced and socially constructed across LMIC contexts. Policymakers who receive a single DALY estimate are therefore receiving the output of a contested normative judgment one that has been partially but not fully democratised presented as an epidemiological fact [15], and subsequent revisions while methodologically improved continue to rely on preferences that do not reflect how disability is experienced and socially constructed in diverse LMIC contexts [16]. A study examining the impact of different disability weighting schemes on the estimated effectiveness of hospital interventions in India found that DALY estimates varied substantially depending on which set of weights was applied, with the choice of weighting scheme materially altering both the magnitude of benefit estimated and, by extension, the cost-effectiveness ranking of the intervention [17]. Policymakers who receive a single DALY estimate are receiving the output of a contested normative judgment presented as an epidemiological fact [15].

## Epistemic injustice and the politics of measurement

The question of who produces global health metrics is inseparable from the question of whose health problems these metrics will illuminate [3]. IHME is based in Seattle, and most institutions that produce health indices, burden of disease estimates, and cost-effectiveness thresholds are located in the Global North [3]. The institutional origins of IHME are instructive here. IHME was founded in 2007 at the University of Washington with a $105 million founding grant from the Bill & Melinda Gates Foundation at the time the largest gift in the university’s history and received a further $279 million from the same foundation in 2017. This creates a structural circularity that deserves explicit acknowledgement: the foundation that funds IHME also uses DALY-based evidence to shape its own grant-making priorities and to evaluate the programmes it funds. When a single philanthropic actor simultaneously finances the production of global disease burden estimates and deploys those estimates to direct billions of dollars of health investment, the governance of global health metrics becomes inseparable from the governance of global health financing itself. This is not an argument against rigorous measurement, but it is an argument for independent, diversified, and democratically governed measurement institutions [18–20].

The research agenda that determines which diseases receive modelling investment, which data sources are treated as authoritative, and which methodological assumptions are considered acceptable is shaped overwhelmingly by institutions in high-income countries, with limited co-production by researchers and policymakers from the settings these estimates are meant to inform [21]. Recent scholarship has described this dynamic as a form of epistemic injustice, a structured exclusion of knowledge produced in LMICs from the frameworks that govern LMIC health policy [22].

The consequences of this extend beyond symbolic recognition [22]. External agenda-setting through metrics shapes not only what is measured but also what is funded and, therefore, what is possible [3]. When donor reporting requirements mandate the reporting of specific DALY-based indicators, health ministries that lack the data infrastructure to generate these indicators face not only technical constraints but also political and financial ones: the inability to speak the language of global health metrics is punished through reduced access to financing [23]. Performance-based financing (PBF) programmes, now operating in over 30 countries across sub-Saharan Africa, formalise this logic by conditioning fund disbursements on the achievement of pre-specified, externally defined indicator targets structurally advantaging settings with stronger data infrastructure regardless of actual population health improvements. Countries in fragile and conflict-affected settings, which face the greatest difficulty generating the required metrics, are therefore penalised twice: they are least able to produce the data donors demand, and least able to access the financing they most urgently need. A systematic review of PBF programmes found that fewer than half of supporting studies had been published in peer-reviewed literature, yet donor agencies continued conditioning billions of dollars of health financing on indicator performance, revealing how the demand for metric compliance precedes and often substitutes for credible evidence of effectiveness. This dynamic has been described, with increasing precision, as a form of data colonialism a system in which the epistemological frameworks of powerful external actors structure the evidence that LMIC institutions are required to produce and the interventions they are permitted to pursue [24]. The construction of success in global health programmes the selection of metrics that emphasize what can be counted, attributes positive trends to funded interventions, and marginalizes systemic shortcomings is not a neutral technical exercise but a deeply political one, as historical analyses of major disease control programmes have repeatedly demonstrated [25].

## Towards context-sensitive and locally driven measurement

**Fig. 1:**
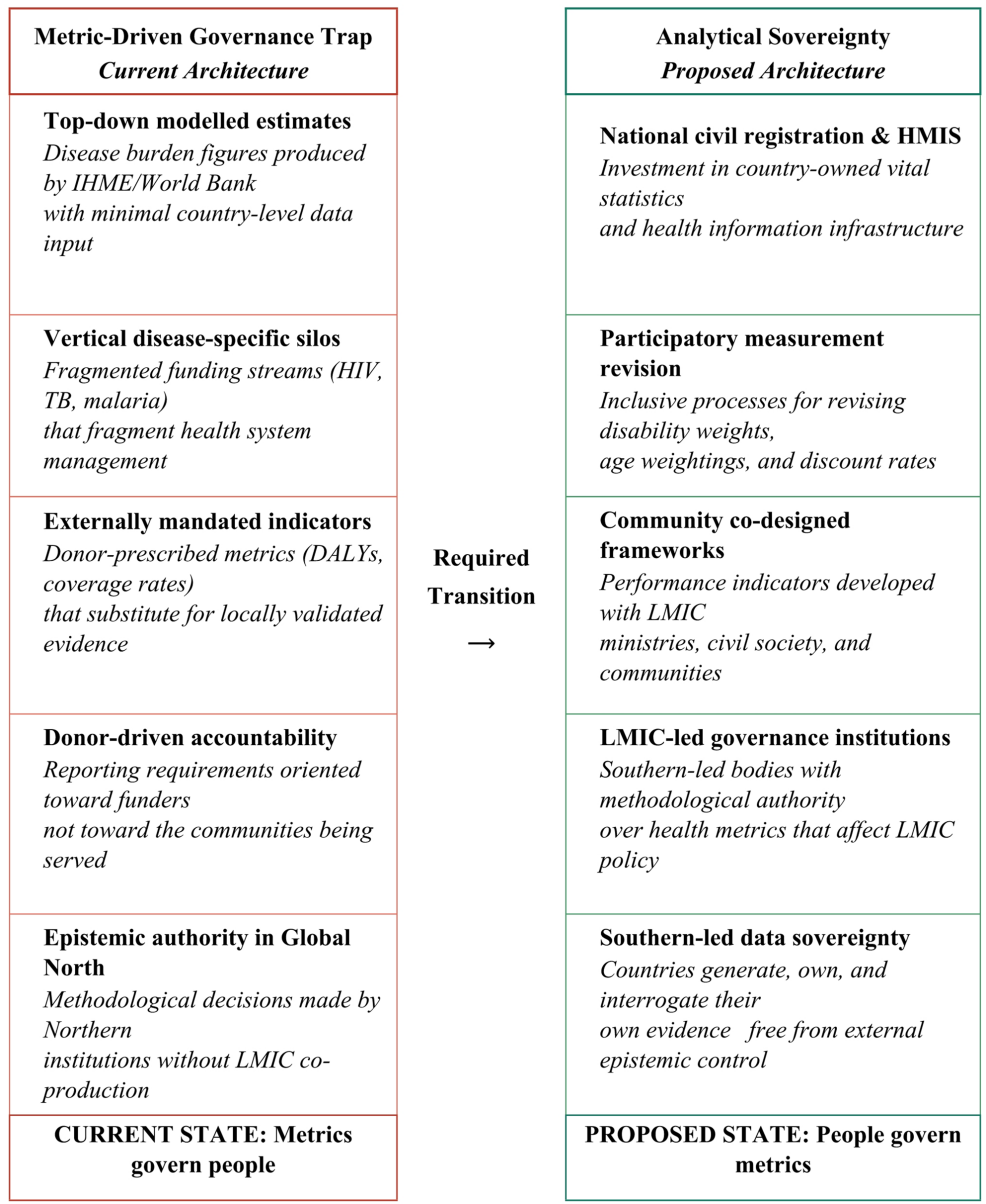
From metric-driven governance trap to analytical sovereignty

None of this implies that systematic measurement is dispensable. It implies, rather, that the current measurement architecture requires profound reform reform that begins not with the refinement of existing metrics but with a fundamental rethinking of their governance, production, and application (Fig. 1) [3]. Several directions are particularly promising.

First, the primary investment in global health measurement should shift from international modelling to national data systems [26]. Strengthening civil registration and vital statistics, health management information systems, and facility-based surveillance in LMICs is not a technical preparation for future metrics; it is a health system strengthening intervention that builds the analytical sovereignty that renders countries capable of generating their own evidence [26]. Africa’s overdependence on externally modelled estimates and the concomitant inability of ministries of health to interrogate or contest those estimates reflect decades of underinvestment in this foundational infrastructure [6].

Second, the disability weights, age weightings, and discount rates embedded in the DALY calculations should be subject to genuinely participatory revision processes that include LMIC populations, disability advocates, and community representatives [15]. The normative assumptions of global metrics are not immutable scientific parameters; they are choices that should be made through inclusive deliberation rather than expert consensus in high-income settings [16]. Amartya Sen’s capability approach developed by the Nobel laureate economist and philosopher Amartya Sen and later extended by Martha Nussbaum holds that human well-being should be evaluated not by income, utility, or the mere presence of resources, but by the substantive freedoms and real opportunities that people have to live lives they have reason to value. In health terms, the approach shifts the evaluative question from ‘did this intervention reduce DALYs?’ to ‘does this person now have a genuine ability to be healthy, move freely, participate in community life, and avoid preventable suffering?’ a reframing that brings social determinants, structural inequities, and community-defined priorities into view rather than reducing them to a single number. This theoretically richer and more contextually flexible framework is increasingly being operationalised in LMIC health evaluation contexts, offering a practical foundation for measurement systems that capture what populations themselves identify as meaningful health offers a theoretically richer and more contextually flexible alternative evaluative framework than utility-based metrics alone and is increasingly being operationalized in LMIC contexts [27].

Third, qualitative and system-level evidence must be institutionally legitimized alongside quantitative metrics [28]. Health system resilience, community trust in services, the quality of patient-provider interaction, and the capacity of a system to absorb and respond to shocks are not captured in coverage indicators or cost-effectiveness ratios, but they are determinants of long-run health outcomes [28]. The development of composite, context-sensitive health system performance frameworksco-designed with LMIC ministries, civil society, and community representativeswould complement rather than replace quantitative measurement while capturing dimensions of health system function that current metrics render invisible [28].

Finally, the governance of global health metrics institutions must be substantially reformed [29]. The concentration of methodological authority in a small number of Northern institutions, combined with the leverage that donors exercise over LMIC reporting requirements, constitutes a structural power asymmetry that no technical refinement of metrics alone can resolve [29]. Increasing the representation of LMIC researchers and policymakers on advisory and governance bodies, redirecting funding towards Southern-led measurement initiatives, and establishing norms of co-production and data sovereignty are preconditions for metrics that genuinely reflect and serve LMIC health needs [30].

## A call for reckoning

The global health community has long acknowledged that data quality in LMICs is inadequate and that metrics must be contextualized. It has been far slower to acknowledge the degree to which inadequate metrics, applied with misplaced confidence, actively harm the populations they are meant to serve. The DALYs, QALYs, and coverage rates that structure global health financing are not neutral instruments; they carry embedded assumptions about what health is worth, whose experiences of illness count, and what kinds of interventions merit investment. When these assumptions diverge systematically from the realities of LMICs when modelled estimates substitute for genuine surveillance, when vertical disease indicators crowd out investments in system function, and when Northern institutions define the terms on which Southern health is evaluated the result is not better policy. It is a mirror that flatters the measurement architecture and obscures the suffering it cannot see.

Global health must move beyond the metric-driven governance trap. Moving money, legitimacy, and methodological authority closer to where illness is actually lived is not an optional refinement; it is a precondition for decisions that reflect, rather than overwrite, LMIC realities. What is presented as evidence-based policy is, in reality, a system that systematically mismeasures and therefore misgoverns health in the very populations it claims to serve. Until this is confronted, global health metrics will remain less a tool of accountability than a technology of abstraction that makes bad decisions look scientifically inevitable.

This conceptual framework illustrates the required directional transition in global health measurement governance. The left panel depicts the current architecture in which top-down modelled estimates, vertical funding silos, and donor-mandated indicators lock LMICs into externally defined priorities. The right panel depicts the proposed shift toward analytical sovereignty through national data systems, participatory measurement processes, and locally driven governance institutions. The transition is not a balance between two equally valid approaches, but a fundamental reorientation of methodological authority closer to where illness is actually lived.

## Data Availability

No datasets were generated or analysed during the current study.
